# Impact of Mixed Equine Influenza Vaccination on Correlate of Protection in Horses

**DOI:** 10.3390/vaccines6040071

**Published:** 2018-10-04

**Authors:** Mohamed Dilai, Mohammed Piro, Mehdi El Harrak, Stéphanie Fougerolle, Mohammed Dehhaoui, Asmaa Dikrallah, Loïc Legrand, Romain Paillot, Ouafaa Fassi Fihri

**Affiliations:** 1 Department of Medicine, Surgery and Reproduction, Hassan II Institute for Agronomy and Veterinary Medicine, Rabat-Institutes 10101, Morocco; vetpiro@yahoo.fr; 2 M.C.I Animal Health—Lot 157, ZI South-West P.O. Box 278, Mohammedia 28810, Morocco; m.elharrak@mci-santeanimale.com; 3 Normandie Univ, UniCaen, Biotargen, 3 rue Nelson Mandela, 14280 Saint-Contest, France; stephanie.fougerolle@laboratoire-labeo.fr (S.F.); loic.legrand@laboratoire-labeo.fr (L.L.); Romain.PAILLOT@laboratoire-labeo.fr (R.P.); 4 LABÉO Frank Duncombe, 1 route de Rosel, 14053 Caen CEDEX 4, France; 5 Department of Statistics and Applied Informatics, Agronomic and Veterinary Institute Hassan II, Rabat-Institutes 10101, Morocco; m.dehhaoui@iav.ac.ma; 6 Department of Microbiology, Immunology and Contagious Diseases, Agronomic and Veterinary Institute Hassan II-B.P 6202 Rabat-Institutes, Rabat-Institutes 10101, Morocco; asmaa.dikrallah@gmail.com (A.D.); fassifihri.ouafaa@gmail.com (O.F.F.)

**Keywords:** equine influenza, vaccination, antibody, SRH, comparison, mixed vaccination

## Abstract

To evaluate the humoral immune response to mixed Equine Influenza vaccination, a common practice in the field, an experimental study was carried out on 42 unvaccinated thoroughbred weanling foals divided into six groups of seven. Three groups were vaccinated using a non-mixed protocol (Equilis^®^ Prequenza-Te, Proteqflu-Te^®^ or Calvenza-03^®^) and three other groups were vaccinated using a mix of the three vaccines mentioned previously. Each weanling underwent a primary EI vaccination schedule composed of two primary immunisations (V1 and V2) four weeks apart followed by a third boost immunisation (V3) six months later. Antibody responses were monitored until one-year post-V3 by single radial haemolysis (SRH). The results showed similar antibody responses for all groups using mixed EI vaccination and the group exclusively vaccinated with Equilis^®^ Prequenza-TE, which were significantly higher than the other two groups vaccinated with Proteqflu-TE^®^ and Calvenza-03^®^. All weanlings (100%) failed to seroconvert after V1 and 21% (9/42) still had low or no SRH antibody titres two weeks post-V2. All weanlings had seroconverted and exceeded the clinical protection threshold one month after V3. The poor response to vaccination was primarily observed in groups exclusively vaccinated with Proteqflu-Te^®^ and Calvenza-03^®^. A large window of susceptibility (3–4.5-month duration) usually called immunity gap was observed after V2 and prior to V3 for all groups. The SRH antibody level was maintained above the clinical protection threshold for three months post-V3 for the groups exclusively vaccinated with Proteqflu-Te^®^ and Calvenza-03^®^, and six months to one year for groups using mixed EI vaccination or exclusively vaccinated with Equilis^®^ Prequenza-Te. This study demonstrates for the first time that the mix of EI vaccines during the primary vaccination schedule has no detrimental impact on the correlate of protection against EIV infection.

## 1. Introduction

Equine influenza (EI) is a major respiratory disease of equids caused by type A influenza viruses. Equine Influenza Virus (EIV) isolates are classified by their subtype and named based on location and year of isolation. Two different subtypes have been designated based on antigenic properties of the haemagglutinin (HA) and neuraminidase (NA) envelope glycoproteins [1]. The H7N7 subtype (A/equine/Prague/1/1956 as prototype strain) was first identified in Eastern Europe in 1956 [1,2], but has not been isolated since the late 1970s and the World Organization for Animal Health (OIE) stipulates that there is no longer a requirement for a representative of this subtype in Equine influenza (EI) vaccines [3]. Equine influenza virus (EIV) of the H3N8 EIV (A/equine/Miami/1/1963 as prototype strain) were first described in 1963 following a major EI epizooty observed in the United States [4], and then diverged at the end of the 1980s into two antigenically distinct lineages, Eurasian and American [5,6]. The American lineage subsequently evolved into the Kentucky, South American, and Florida sub-lineages [1,6], with the Florida sub-lineage dominating for a decade. In the last few years, the Florida sub-lineage diverged into two clades (Florida clade 1 and clade 2; FC1 and FC2, respectively), which contain most of EIV isolated worldwide [1,7,8].

Clinical signs of EI in naïve and unprotected animals are characterised by pyrexia, a serous nasal discharge and persistent cough that allow rapid spreading of the disease by dissemination of infectious aerosols [1,9]. In a susceptible population, morbidity can be as high as 100%; mortality is rare but occurs occasionally in foals that have not acquired maternal antibodies and donkeys. In adults, mortality is generally a consequence of poor health condition and/or secondary bacterial infection [1,10,11].

The economic losses caused by EI epizootics may be substantial, notably by stopping horse races and commercial exchange [9]. Regretfully, this was particularly well illustrated during the 2007 Australian EI outbreak, when around 76,000 horses were infected and the overall cost to control and eradicate the disease from Australia reached AU$1 billion [12,13].

Vaccination against EIV remains to this day one of the most effective methods to prevent or limit the impact of EI outbreaks [14]. Today, EI vaccines commercialized worldwide could be differentiated into three groups based on their technology (i.e., whole inactivated/sub-unit ISCOM-Matrix or ISCOM, viral-vector based and live-attenuated) [1]. Table 1 classes EI vaccines by technologies. 

The whole inactivated EIV vaccines were the first type of vaccine to be developed and were the predominant type of EI vaccine available for decades [1]. The immuno-stimulating complex (ISCOM) technology regroups the ISCOM-based and the ISCOM-Matrix adjuvanted vaccines, which differ in terms of formulation. This technology is based on the particulate ISCOM-Matrix adjuvant, which contains phospholipids, cholesterol and Quil-A (Quillaja) saponin (matrix component) [1,15,16]. ISCOM-based vaccines contain hydrophobic antigen and/or membrane proteins that are directly mixed with the matrix component to give stable, self-adjuvating particles, held together by hydrophobic interactions [1,17]. ISCOM-Matrix-based vaccine contains already prepared matrix particles mixed with purified antigens or whole inactivated pathogens. Immunisation with the ISCOM-matrix-based EIV vaccine stimulated significant EIV-specific single radial haemolysis (SRH) and EIV-specific IFN-gamma synthesis antibody, which support stimulation of cell-mediated immunity (CMI) after immunisation [18]. Modified live EIV vaccine (MLV) contains a live attenuated EIV that retains its ability to infect the host and their immunogenicity. This type of vaccine stimulates a long lasting immune response, involving both antibodies and CMI. This type of vaccine has been marketed only in the USA for over a decade (Flu Avert^®^ I.N. Vaccine) [1]. The principal concerns about the MLV vaccines are reversion to virulence, and safety in pregnant or immunocompromised animals [19]. A recombinant canarypox live virus vaccine for EIV has been widely commercially available since 2003. This vaccine utilizes Carbomer (polyacrylic acid) adjuvant and expresses HA of two EIV H3N8 strains (originally representing the American and Eurasian lineages, since updated with Florida clade 1 and clade 2 strains) [19,20,21]. The stimulation of cell-mediated immunity (CMI) after immunization with a recombinant canarypox vaccine was also demonstrated when naïve horses vaccinated showed an increase in EIV specific IFN-gamma and IL-2 mRNA expression [22].

Vaccine strains update is essential to optimise the chances of preventing vaccine breakdown. The current recommendation of the OIE expert surveillance panel supports inclusion of both FC1 and FC2 representative EIV strains in EI vaccines [3,23,24]. For most EI vaccines, the vaccination schedule consists of a primary course of two immunisations (V1 and V2), 4–6 weeks apart [1], followed by a third (boost) immunisation (V3), 5–6 months later and subsequent annual or bi-annual immunisations according to manufacturer’s recommendations and/or risk of contact with infected animals. It is also important to note that most EI vaccines have been shown to stimulate EIV-specific cell-mediated immunity, which is likely to play a role in protection against EIV infection and/or in the mitigation of clinical signs and virus shedding following EIV infection [1]. The kinetics of the EI vaccines response is well documented [25,26,27,28]. A positive correlation between SRH antibody titres and the level of protection against equine influenza has been demonstrated in several studies. Horses with SRH antibody titres below 85 mm^2^ (clinical protection threshold) are considered insufficiently protected clinically and present a real risk of spreading the disease. Sera with SRH antibody titre between 85 and 120 mm^2^ are considered protected against clinical signs of EI. Horses with SRH antibody titres between 120 abd 154 mm^2^ or more are considered protected against virus shedding (virological protection threshold). Sterilising immunity, which prevents EIV infection, clinical signs of disease and seroconvertion, is rare and requires high SRH antibody levels at the time of infection with EIV [1,24,29]. While the vaccination schedule is similar between EI vaccines, there are no recommendations or restrictions regarding the use of different EI vaccines during the immunisation schedule (i.e., mixed-regime). This practice is not uncommon in racehorses. However, little or no information is available concerning the immunological impact of mixing different EI vaccine brands and their compatibility, which is a concern for some practitioner veterinarians [8]. It has been suggested that the mix of different EI vaccines may have a negative impact on vaccine efficacy [30,31].

The present study aimed to evaluate for the first time the impact of mixing EI vaccines. The specific objective was to measure antibody level using single radial haemolysis (SRH) assay after primary and booster vaccination of 42 naïve thoroughbred weanling foals with mixed and non-mixed EI vaccination regime.

## 2. Materials and Methods

### 2.1. Animals

Eligibility criteria, setting, location and sample size: This study was carried out on a population of 42 unvaccinated thoroughbred weanlings on a stud farm situated 98 km north of Rabat in Morocco (Latitude: 34.5197222 and Longitude: −6.3236111). The weanlings were all born in 2015 and were from mares vaccinated with Calvenza-03^®^. The age at the time of first vaccination (V1) ranged from 143 to 246 days (202 ± 5.2 days). They were randomly divided into 6 groups of 7 animals (block randomization) and kept at the same location for the duration of the study, with no difference in terms of husbandry and care. Sample size (7 weanlings per group) was based on the European Pharmacopoeia criteria for EI vaccine (inactivated), which requires no fewer than 6 horses for the vaccinated group [32]. All animal work obtained owner consent and received ethical approval from the Hassan II Institute for Agronomy and Veterinary Medicine on the 8 of September 2015 (ID project: 1467).

### 2.2. Vaccines

Investigational veterinary products (IVP): Three commercial EI vaccines (among the EI vaccines mostly used in Morocco) were selected: (i) a whole inactivated, ISCO-matrix adjuvanted EI vaccine (Equilis^®^ Prequenza-TE; MSD Animal Health (lot number: A212001)); (ii) a recombinant canarypox-based EI vaccine (Proteqflu-TE^®^; Boehringer Ingelheim, formerly Merial Animal Health (lot number: L419496)), both vaccines were combined with tetanus; and (iii) a whole inactivated EI vaccine combined with equine herpes virus type 1 (EHV-1), (Calvenza-03^®^; Boehringer Ingelheim (lot number: 326-063B)). The EIV strain/antigen composition and adjuvant of each of the three vaccines used in this study are presented in Table 1.

### 2.3. Vaccination Protocol

Intervention and duration of the study: Each weanling received two immunisations (V1 and V2; primary vaccination), 4 weeks apart (Week 0 and Week 4) followed by a third dose (V3; Week 28) 6 months later via deep intra-muscular injection in the left neck using 21G × 1½″, 0.8 × 40 mm needle. Three groups of animals (Groups #1–#3) were administered with a non-mixed EI vaccine regimen, while the other three groups (Groups #4–#6) received a mixed EI vaccine regimen, as presented in Table 2. Out of the 6 EI vaccine combinations possible and in the limit of resources available to conduct this study, 3 combinations were randomly selected. The study was initiated in September 2015 (first immunisation) and lasted until April 2017 (last sampling). This report of clinical trials follows the CONSORT 2010 guidelines (Table S1 and Chart S1 in Supplementary Materials) [33,34].

### 2.4. Collection of Samples

Whole blood samples were collected from animals until one year after V3 at eighteen different occasions, as shown in Table 3. At the time of V1, serum samples were collected to evaluate the presence of maternally-derived antibodies (S1); two weeks after V1 (S2) and at the time of V2 (S3) to measure any onset of immunity after the first immunisation; two weeks after V2 to measure the antibody response at the peak of immunity (S4); 4–24 weeks after V2 to follow the kinetics of humoral response during the primary vaccination (S5–S12) and to measure potential immunity gaps; and finally 4–48 weeks after V3 to determine the duration of humoral immunity after the boost immunisation (S13–S18). All samples were collected by jugular vein puncture on sterile dry tube (without anticoagulant). The sera were extracted by centrifugation and stored at −20 °C until the serological analysis.

### 2.5. Serology

Outcome measurement: Antibodies against FC2 EIV strain A/equine/Richmond/1/07 (H3N8) were measured using single radial haemolysis assay (SRH) according to the OIE recommendations [3]. The A/equine/Richmond1/07 (H3N8) strain was isolated in the United Kingdom in 2007 from the surveillance network of the Animal Health Trust (network financed by the Horserace Betting Levy Board). This EIV strain is representative of the FC2 sub-lineage. FC2 EIV strains are currently circulating in Europe [35] and were also isolated in North Africa [36], which motivated selection of this EIV strain as SRH antigen. As SRH assay require a large amount of viral antigen, this FC2 strain was produced on embryonated chicken eggs “Specific Pathogen Free” at the MCI Animal Health’s laboratory in Mohammedia, Morocco. The antisera reference standard (reference EU SA/4/03 Y0000712) against A/equine/South Africa/4/03 (H3N8) from the European Directorate for the Quality of Medicines and Healthcare (EDQM) was used on each plate as a control. The haemolytic zones resulting from the lysis of the sensitised sheep red blood cells coupled to EIV and guinea pig complement by the antibody in the test sera were measured with a digital calliper. The area of haemolysis was calculated and results were expressed in mm^2^. Analyses were repeated for 33% of samples to confirm reproducibility. Due to limited operator resource available, the study was not masked. However, to limit bias, samples were identified based on the weanling ID, with no indication of the group they belong to until final result analysis.

### 2.6. Statistical Analysis

Statistical analysis was carried out using the IBM SPSS software (Statistical Package for the Social Sciences). The Analysis of variance test (ANOVA) has been used to compare the serology results between each group and the sampling time followed by post hoc test using Tukey’s honest significant difference (HSD). Tests of significance were carried out at the α = 5% level. Microsoft Excel was used for data recording.

## 3. Results

### 3.1. Maternally Derived Antibodies (MDA)

At the time of the first vaccination (V1), only one weanling belonging to Group #2 had detectable SRH antibodies (43 mm^2^). This foal was vaccinated with Proteqflu-TE^®^ (non-mixed protocol) with no evidence of seroconversion after V1 and V2 (41.7 mm^2^ 4 weeks post V2) but a clear response to the third dose of vaccine (V3) (153 mm^2^ four weeks after V3).

### 3.2. SRH Antibody Response

**Figure 1:**
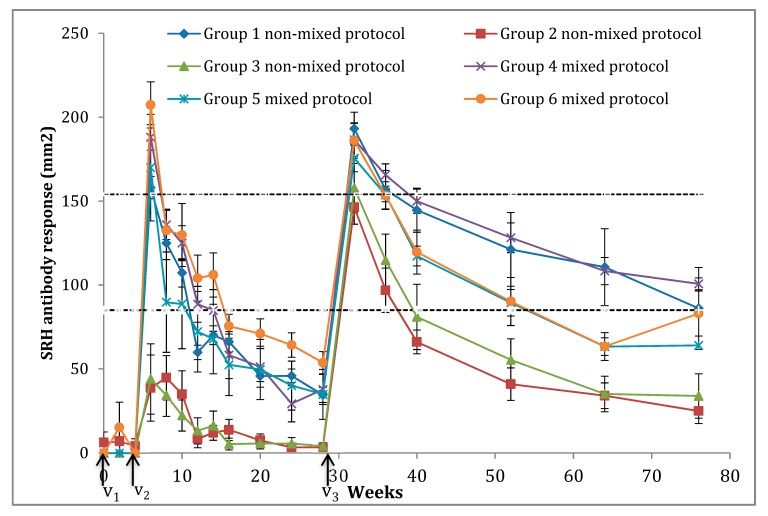
Mean SRH antibody in vaccinated weanlings measured against A/equine/Richmond/1/07. Broken lines represent SRH antibody level 85 mm^2^ and 154 mm^2^ correlating with clinical and virological protection, respectively. The vaccination schedule was carried out as follows: V1, 0 weeks; V2, 4 weeks; V3, 28 weeks. Each group was vaccinated as follows: Group 1, Exclusively Prequenza-TE^®^; Group 2, Exclusively Proteqflu TE^®^; Group 3, Exclusively Calvenza-03^®^; Group 4, V1: Prequenza-TE^®^/V2: Proteqflu-TE^®^/V3: Calvenza-03^®^; Group 5, V1: Proteqflu-TE^®^/V2: Prequenza-TE^®^/V3: Calvenza-03^®^; and Group 6, V1: Calvenza-03^®^/V2: Prequenza-TE^®^/V3: Proteqflu-TE^®^.

Overall, the kinetics of SRH antibody response to the FC2 EIV strain A/equine/Richmond/1/07 (H3N8) were similar for four groups (all mixed EI vaccines groups (#4–#6) and Group #1; non-mixed protocol using Equilis^®^ Prequenza-TE). The SRH antibody responses for Groups #2 and #3 (non-mixed protocols with Proteqflu-TE^®^ and Calvenza-03^®^, respectively) were significantly lower (*p* < 0.05) when compared with other groups. Both groups showed a significantly lower antibody response at 11 sampling time points after V2 and V3 (6, 8, 10, 12, 14, 16, 20, 28, 36, 40, and 52 weeks). The SRH antibody levels measured for each group are presented in Figure 1 and Table 3.

Only one weanling from Group #6 seroconverted after the first immunisation (V1) with a SRH titre of 105 mm^2^ two weeks after V1. At V2, no foals had detectable SRH antibody titre except one with MDA at V1 (29.6 mm^2^). Two weeks post V2, Groups #1, #4, #5 and #6 responded well to the vaccination without significant difference between them (*p* > 0.05) (158 ± 6.6 mm^2^, 188 ± 7.6 mm^2^, 169.9 ± 31.7 mm^2^ and 207.3 ± 13.7 mm^2^, respectively). The virological protection threshold (120–154 mm^2^) was reached for these four groups. For Groups #2 and #3, SRH antibody levels were on average below the 85 mm² threshold for clinical protection and was significantly lower (*p* < 0.05) than the other four groups (38.5 ± 19.7 mm^2^ and 44 ± 21 mm^2^, respectively). The mean SRH antibody titre declined under the clinical protection threshold two months post V2 for Group #1 (59.8 ± 4.3 mm^2^), twn weeks post V2 for Group #4 (84.8 ± 12.4 mm^2^), two months post V2 for Group #5 (72.1 ± 24 mm^2^) and three months post V2 for Group #6 (75.4 ± 7.1 mm^2^). Six months post V2 (i.e., the time of V3), all groups showed low SRH antibody levels and especially Groups #2 and #3 (34.4 ± 4.5 mm^2^ for Group #1, 3.4 ± 2.3 mm^2^ for Group #2, 4.2 ± 2.1 mm^2^ for Group #3, 37.7 ± 9 mm^2^ for Group #4, 34.8 ± 14.9 mm^2^ for Group #5 and 53.6 ± 6.6 mm^2^ for Group #6). Four weeks after V3 (Week 32), a strong SRH antibody response was measured in all groups, with titres exceeding the virological protection threshold (with the exception of one horse in Group #2). The only significant difference (*p* < 0.05) at this time point was between Group #1 (193.1 ± 9.8 mm^2^) and Group #2 (146.3 ± 10.1 mm^2^). Then, the immune response declined rapidly and significantly under the clinical protection threshold three months post V3 (Week 40) for Groups #2 and #3 (66 ± 7.1 mm^2^ and 81 ± 19.4 mm^2^, respectively) to reach a very low SRH antibody levels (24.9 ± 7.5 mm^2^ and 33.8 ± 13.2 mm^2^, respectively) one year after V3 (Week 76). For Groups #5 and #6, SRH antibody levels declined but remained above the clinical protection threshold when measured six months post V3 (Week 52; 89.4 ± 7.9 mm^2^ and 90.1 ± 14.3 mm^2^, respectively) and reached SRH antibody titres of 64 mm^2^ and 83 ± 13.4 mm^2^ one year post V3 (Week 76), respectively. Groups #1 and #4 SRH antibody levels declined after V3 but remained above the clinical protection threshold and reached 86 ± 24.4 mm^2^ and 100.7 ± 3.5 mm^2^ one year after V3, respectively.

## 4. Discussion

This study compared the kinetics of humoral immunity after prime and boost vaccination of 42 naïve weanlings using mixed and non-mixed EI vaccine protocols. Based on SRH antibody levels, there was no difference between groups using mixed EI vaccines and the group vaccinated exclusively with Equilis^®^ Prequenza-TE. The SRH antibody levels of these groups were significantly higher than titres measured in two other groups that received non-mixed EI vaccine protocol (i.e., Proteqflu-TE^®^ and Calvenza-03^®^).

Only one EI vaccine (Proteqflu-TE^®^) contains the representative FC2 EIV strain A/equine/Richmond/1/07 (H3N8); homologous strain used in this study as SRH antigen. The other two EI vaccines (Equilis^®^ Prequenza-TE and Calvenza-03^®^) contain H3N8 heterologous strains. The cross protection between EIV strains is well documented, as recently demonstrated by Paillot et al. (2018) [37]. Gildea et al. (2011) [25] found that the humoral antibody response was similar for all vaccines used and, for all antigens tested [25], sera from vaccinated horses were cross-reactive [38]. Paillot et al. (2013) demonstrated that the vaccination with non-updated inactivated EI and tetanus vaccine containing European and American lineage developed a cross-reactive SRH antibody response against A/equine/Richmond/1/07 [39].

Two of the three EI vaccines products used in this study were combined with the tetanus toxoid (Table 1) to get closer to field conditions. There is no evidence that the presence of tetanus toxoid compromises the response to EI vaccination [40]. The use of different EI vaccines in this study does not allow identifying an impact of the presence of tetanus toxoid on the SRH antibody response.

At the time of V1, the mean age of the weanlings was 6.7 ± 0.17 months and only one weanling in this study had detectable SRH antibodies (43 mm^2^), likely to be of maternal origin in the absence of respiratory disease history and contact with EIV. In a similar study of unvaccinated weanlings (5–10 months of age), 92% of animals were seronegative for H3N8 EIV as measured by SRH [25].

In the 1989 UK EI epizootic, animals with SRH antibody levels below 50 mm^2^ were found to be 15 times more susceptible to EIV infection than those above 50 mm^2^ [25,41]. In the current study, 100% of the weanlings failed to seroconvert after V1. Two weeks post V2, 21% (9/42) of the weanlings still had SRH antibody levels below 50 mm^2^ (with five foals that failed to seroconvert and had no measurable SRH antibody). The poor responders were mostly observed in groups vaccinated with Proteqflu-Te^®^ and Calvenza-03^®^ (4/7 in each group) and failed to seroconvert or developed low SRH antibody titre until the third immunisation. One month after V3, all weanlings seroconverted and had exceeded the clinical protection threshold. Such a high incidence of poor responders after V2 has been rarely reported in previous experimental studies. Gildea et al. (2011) [25] described 43% of poor responders after V1 and 7% post-V2. In a study conducted by Fougerolle et al. in 2016 [14] on thoroughbred foals vaccinated with Proteqflu-Te^®^, 56.8% of vaccinated animals were found to have no detectable SRH antibodies two weeks post V2, a poor response to vaccination related to the presence of MDA and an early age at the time of V1. It has been demonstrated as several occasions in experimental vaccination trial that animals of one year or more are likely to seroconvert after V1. Paillot et al. (2008) [42] reported that only one of the six EI vaccinated animals (ISCOM-based EI vaccine) did not possess detectable SRH antibodies after the first vaccination. Bryant et al. (2010) [43] showed that all animals vaccinated with Proteqflu-Te^®^ seroconverted 14 days post-V1. In the current study, the age at V1 was not significantly different between groups (*p* = 0.18). However, the average age in Groups #2 and #3 (189 ± 15 and 180 ± 12 days, respectively) was lower than the other groups (206 ± 9, 222 ± 7, 214 ± 11 and 202 ± 17 days for Groups #1, #4, #5 and #6, respectively).

In this study, the serological response induced by the ISCOM-matrix vaccine (Equilis^®^ Prequenza-TE) was superior to the ones stimulated by the recombinant canarypox-based EI vaccine Proteqflu-Te^®^ and the multivalent whole inactivated vaccine (Calvenza-03^®^). Additionally, the inclusion of an ISCOM-matrix equine influenza (EIV) vaccine induced the most durable antibody responses when used exclusively or mixed with other EI vaccines. Some previous studies have suggested that monovalent EI vaccines are more effective than multivalent EIV/EHV vaccines [44,45]. However, these results were different with those obtained by Gildea et al. (2011) [25] and Gildea et al. (2013) [27] when they described the same pattern of antibody response obtained with Equilis^®^ Prequenza-Te^®^ and Proteqflu-TE^®^ and demonstrated that the multivalent EI/EHV vaccine (Equilis Resequin) stimulated a comparable humoral immune response to one induced by monovalent EI vaccine [46]. At the current time, no obvious factor may explain the difference in response measured between Groups #1 to #3.

A large window of susceptibility (i.e., immunity gap) was observed after V2 and prior to V3 for all groups due a decline of antibody titre induced by primary immunisations. The poor durability of the immune response after primary EI vaccination has been demonstrated previously [8,25,47]. The SRH antibody levels measured in weanlings with Equilis^®^ Prequenza-Te were similar to the antibody response measured in ponies vaccinated with the same EI vaccine in a recent study conducted by Paillot et al. (2018) [37]. Significant clinical and virological protections were measured when these ponies were experimentally infected with a recent FC2 EIV strain, five months after V2 [37].

Overall and throughout the trial, the three groups vaccinated using a mixed EI vaccine protocol (Groups #4–#6, Table 2) developed a good humoral immune response after primary and boost immunisations, which was comparable to the response measure in weanlings exclusively vaccinated with Equilis^®^ Prequenza TE and similar to those described and obtained in previous studies [8,18,23,26,27,48]. In this study, four weeks after V3, all the weanlings reached the virological threshold of protection. The decline in SRH antibody titre depended on the group. The SRH antibody titres were below the clinical protection threshold 2–3 months post-V3 for Groups #2 and #3, six months post-V3 for Groups #5 and #6, and still above this threshold one year post-V3 for Groups #1 and #4. It is interesting to note the significant difference in mean titres between Groups #4 and #5 at Week 64, and the observable trend towards significance from Week 40. However, both Groups #4 and #5 had very similar mean titres both before and after V3 (Weeks 28 and 32). While both groups received the same vaccine at V3, the difference of decay could be linked to the vaccine used at V1 (Group #4 and #5 received Equilis^®^ Prequenza-TE and ProteqFlu-TE^®^ at V1, respectively). One possible explanation for the accelerated decay of the SRH antibody response after V3 measured for Group #5, when compared with Group #4, would be a sub-optimal priming induced by V1. This hypothesis is supported by the kinetics measured for Group #2 (ProteqFlu-TE^®^ only) that tends to indicate a poor priming (low response after V2). The SRH antibody kinetics measured after V3 for Group #6 (a group that received Calvenza-03^®^ at V1) is similar to Group #5. The SRH kinetics measured for Group #3 (Calvenza-03^®^ alone) also tends to indicate a poor priming (low response after V2). This difference measured between Groups #4 and #5 clearly tends to indicate that the first EI vaccine administered (i.e., V1) will strongly influence the response after at least two subsequent boost immunisations.

While protection against EI induced by modern vaccines is not solely based on an antibody response but is likely to also involve stimulation of cell-mediated immunity, as demonstrated for several EI vaccines [18,42,49], these results suggest that the claim of one-year duration of protective immunity after V3 may require careful evaluation [25]. In a similar study, Newton et al. (2000) [30] identified a high-risk window approximately four months following V3 and suggested that a program of three doses followed by annual booster vaccination dose may not always provide sufficient protective immunity.

These findings can answer some questions asked during the annual meeting of the French Equine Veterinarians Association (AVEF) at Reims (France) in 2016, where Paillot et al. (2017) pointed out that little information is available on the immunological impact induced by mixing different EI vaccine brands and the scientific evidence is sparse [8]. In the absence of experimental data regarding the consequence of mixing EI vaccines, some sero-epidemiological studies can provide some information. Ryan et al. (2015) [31] conducted an observational field study in Ireland, where 102 vaccinated thoroughbred horses in training were sampled one month after booster vaccination with ProteqFlu-Te^®^. The majority of horses (95%) received more than one EI vaccine product while 32% had received three vaccine brands. Furthermore, a superior antibody response was observed among horses that did not receive the same vaccine product for the first three doses of primary vaccination [31]. In 2003, the EI outbreak in Newmarket revealed that the risk of infection with EIV was significantly reduced in animals with a mixed vaccine history. A whole inactivated EI vaccine adjuvanted with hydroxide aluminium seems to have provided limited protection during this outbreak, which in part explains the benefit of mixed vaccination [8,50]. In Hong Kong, the vaccination of EI is obligatory for imported horses. Upon arrival, horses receive a fresh primary course of EI immunization with a unique EI vaccine. Due to numerous countries of origin and the diversity of EI vaccines used worldwide, the mix of EI vaccine is inevitable. The preliminary results demonstrated that horses with mixed EI vaccine history developed a good SRH antibody response after arrival and mandatory EI immunization with Equilis^®^ Prequenza TE [8,51]. Another study from a pharmaceutical company demonstrated that a primary immunisation with the live attenuated EI vaccine Flu Avert IN (administrated intra-nasaly), followed one month later by a second dose of the EI vaccine Equilis Prequenza^®^ (administered intra-muscularly), induced a robust immune response that was superior to the one obtained after primary and secondary immunisations with the Flu Avert vaccine alone [8,52].

## 5. Conclusions

In summary, this study represents the first demonstration in field condition that the practice of mixing EI vaccines during the primary vaccination schedule had no detrimental effect on the SRH antibody response, a well-described correlate of protection against EIV infection. Weanlings with mixed EI vaccination protocols developed similar humoral antibody responses to weanlings exclusively vaccinated with Equilis^®^ Prequenza-Te. The inclusion of an ISCOM-matrix-based equine influenza (EIV) vaccine induced the most durable antibody responses when used exclusively or mixed with other EI vaccines. Results tend to indicate that clinical practice in Morocco could be improved by the inclusion of this type of EI vaccine to the vaccination regimen. The humoral immunity induced by the recombinant canarypox-based EI vaccine Proteqflu-Te^®^ and the multivalent vaccines Calvenza-03^®^ EIV/EHV was significantly lower, with no obvious reasons that could explain this result. The immune response kinetics confirmed a large window of susceptibility to EIV infection (i.e., immunity gap) between V2 and V3. The duration of immunity after V3 was dependent of the EI vaccine used. Bi-annual boost immunisation rather than annual vaccination may need to be considered in risk of increased contact with other and potentially infected animals.

## Figures and Tables

**Table 1:** Equine Influenza vaccines classed by technologies.

Technology	Study	Vaccine/Manufacturer	Nature	Adjuvant	Compositions
Whole inactivated/Sub-unit ISCOM/ISCOM-Matrix	Used	Equilis^®^ Prequenza-TE (MSD Animal Health)	Whole inactivated	ISCOMatrix, chol., P. saponin, Phos. choline.	-A/Equi-2/South Africa/4/03 -A/Equi-2/Newmarket/2/93 -Anatoxine tétanique
	Used	Calvenza-03 EIV/EHV^®^ (Boehringer Ingelheim)	Whole inactivated	Carbimmune	-A/Equi-2/Newmarket/2/1993 -A/Equi-2/Kentucky/1995 -A/Equi-2/Ohio/2003 -EHV 1 souche KyA
	Not used	Duvaxyn IE-T^®^ (Elanco Animal Health)	Whole inactivated	Carbomer, Alum. Hydr.	-A/Equi-1/Prague/56 (H7N7) -A/Equi-2/Newmarket 1/93 -A/Equi-2/Suffolk/89 -Anatoxine tétanique
	Not used	Equip-FT^®^(Pfizer)	Subunit	ISCOM, Quillaic Acid derivative, Aluminium phosphate	-A/Equi-1/Newmarket 77 -A/Equi-2/Borlange 91 -A/Equi-2/Kentucky 98 -Anatoxine tétanique
Viral-vector based	Used	Proteqflu-TE^®^ (Boehringer Ingelheim)	Recominant canarypox	Carbomer	-A/Eq/Ohio/03 -A/Eq/Richmond/1/07 -Anatoxine tétanique
Modified live EIV	Not Used	Flu Avert^®^ I.N. (MSD Animal Health)	whole virus	Not applicable	Attenuated, cold adapted EIV: Kentucky/91 (H3N8)

**Table 2:** Vaccination protocol per group.

Weanling Groups	Number of Weanlings	Vaccination Protocol
Group #1	7	V1: Prequenza-TE^®^ V2: Prequenza-TE^®^ V3: Prequenza-TE^®^
Group #2	7	V1: Proteqflu-TE^®^ V2: Proteqflu-TE^®^ V3: Proteqflu-TE^®^
Group #3	7	V1: Calvenza-03^®^ V2: Calvenza-03^®^ V3: Calvenza-03^®^
Group #4	7	V1: Prequenza-TE^®^ V2: Proteqflu-TE^®^ V3: Calvenza-03^®^
Group #5	7	V1: Proteqflu-TE^®^ V2: Prequenza-TE^®^ V3: Calvenza-03^®^
Group #6	7	V1: Calvenza-03^®^ V2: Prequenza-TE^®^ V3: Proteqflu-TE^®^

**Table 3:** SRH antibody titres per group and sampling date (average and standard error, titres expressed as mm²).

Weeks	Vaccination	Sampling	Groups											
			#1		#2		#3		#4		#5		#6	
			Average	SE	Average	SE	Average	SE	Average	SE	Average	SE	Average	SE
0	V1	S1	0	0	6.2	6.2	0	0	0	0	0	0	0	0
2		S2	0	0	6.9	6.9	0	0	0	0	0	0	15	15
4	V2	S3	0	0	4.2	4.2	0	0	0	0	0	0	0	0
6		S4	158	6.6	38.5	19.7	44	21	188	7.6	169.9	31.7	207.3	13.7
8		S5	124.9	9.8	44.7	13.2	34.2	12.6	135.9	8.7	89.7	29.9	132.5	12.8
10		S6	107.2	8.4	34.9	13.9	22.2	9.3	124.9	10.4	88.6	26.6	129.8	18.8
12		S7	59.8	4.3	8.3	5.2	13.1	7.7	88.5	10.7	72.1	24	104	13.8
14		S8	70	5.6	11.9	4.3	16.1	8.7	84.8	12.4	67.8	20.7	106	13.1
16		S9	66.1	6.1	13.6	6.2	5.3	3.5	58.2	14	52.4	18.4	75.4	7.1
20		S10	45.7	3.3	7.4	3.8	5.6	3.3	51.2	12.2	49.6	17.9	71	8.8
24		S11	45.8	4.1	3.3	2.4	5.5	3.7	29.2	10.8	40.0	14.6	64.2	7.3
28	V3	S12	34.4	4.5	3.4	2.3	4.2	2.1	37.7	9	34.8	14.9	53.6	6.6
32		S13	193.1	9.8	146.3	10.1	157.8	14.7	186.5	9.8	175.5	8.1	186.1	10.5
36		S14	156.8	11.5	96.8	13.3	114.7	15.6	165.5	6.7	153.7	4	153.3	8.3
40		S15	144.6	13.2	66	7.1	81	19.4	150	7.2	117.2	5.9	119.6	13.1
52		S16	121.2	22.0	40.9	9.7	55.3	12.6	128.2	8.9	89.4	7.9	90.1	14.3
64		S17	110.6	22.9	34	7.6	35	10.6	108.3	8.1	63.2	5.3	63.3	8.3
76		S18	86	24.4	24.9	7.5	33.8	13.2	100.7	3.5	64.0		83	13.4

No significant difference was measured after V3 regarding the mean SRH antibody response for four groups (#1, #4, #5 and #6), except at Week 64, where the SRH antibody response was significantly higher (*p* < 0.05) in Group #1 and #4 (110.6 ± 22.9 mm^2^, 108.3 ± 8.1 mm^2^, respectively) when compared with Group #5 and #6 (63.2 ± 5.3 mm^2^ and 63.3 ± 8.3 mm^2^, respectively).

## Supplementary Materials

The following are available online at http://www.mdpi.com/2076-393X/6/4/71/s1, Table S1: CONSORT 2010 checklist of information to include when reporting a randomised trial. Chart S1: CONSORT Flow Diagram.
