# Supplementary material for: Impact of Mixed Equine Influenza Vaccination on Correlate of Protection in Horses
Source: Vaccines (Basel). 2018 Oct 4;6(4):71. doi: 10.3390/vaccines6040071 (PMC6313876; doi:10.3390/vaccines6040071)
Supplement: Supplementary file 1 [file vaccines-06-00071-s001.zip › vaccines-347927-SI/Supplementary_Chart S1 Dilai et al.doc]

**Allocation**

**Enrollment**

**Follow-Up**

**Follow-Up**

**Follow-Up**

**Follow-Up**

**Follow-Up**

**Follow-Up**

Assessed for eligibility **(n= 42)**

**Gr#1:** Allocated to intervention **(n= 7)**

-Received allocated intervention **(n=7)**

-Did not receive allocated intervention (give reasons) **(n=0)**

Randomized **(n= 42)**

**Gr#2:** Allocated to intervention **(n= 7)**

-Received allocated intervention **(n=7)**

-Did not receive allocated intervention (give reasons) **(n=0)**

**Gr#3:** Allocated to intervention **(n= 7)**

-Received allocated intervention **(n=7)**

-Did not receive allocated intervention (give reasons) **(n=0)**

**Gr#4:** Allocated to intervention **(n= 7)**

-Received allocated intervention **(n=7)**

-Did not receive allocated intervention (give reasons) **(n=0)**

**Gr#5:** Allocated to intervention **(n= 7)**

-Received allocated intervention **(n=7)**

-Did not receive allocated intervention (give reasons) **(n=0)**

**Gr#6:** Allocated to intervention **(n= 7)**

-Received allocated intervention **(n=7)**

-Did not receive allocated intervention (give reasons) **(n=0)**

**Chart S1: Impact of mixed equine influenza vaccination on correlate of protection in horses**

Excluded **(n=0)**

♦  Not meeting inclusion criteria **(n=0)**

♦  Declined to participate **(n=0)**

♦  Other reasons **(n=0)**

**Gr#3:** Lost to follow-up **(n= 2):**

- Foals sold by the owner at 40 and 76 weeks of the trial.

- Discontinued intervention **(n=2):**

Sometimes, it’s difficult to catch the foals and take a blood samples

**Gr#2:** Lost to follow-up **(n= 3):**

-1 foal sold by the owner at 40 weeks and 2 foals at 76 weeks of the trial.

- Discontinued intervention **(n=4):**

Sometimes, it’s difficult to catch the foals and take a blood samples

**Gr#1:** Lost to follow-up **(n= 3):**

- Foals sold by the owner at 36, 64 and 76 weeks of the trial.

- Discontinued intervention **(n=2):**

Sometimes, it’s difficult to catch the foals and take a blood samples

**Gr#4:** Lost to follow-up **(n= 4):**

- 3 foals sold by the owner at 76 weeks of the trial.1 foal dead at 16 weeks of the trial following a paddock accident.

- Discontinued intervention **(n=2):**

Sometimes, it’s difficult to catch the foals and take a blood samples

**Gr#5:** Lost to follow-up **(n= 6):**

- 1 Foal sold by the owner at 64 weeks and 4 foals at 76 weeks of the trial. 1 foal dead at 10 weeks of the trial following a paddock accident

- Discontinued intervention **(n=0)**

**Gr#6:** Lost to follow-up **(n= 4):**

- Foals sold by the owner at 40, 64 and 76 weeks of the trial.

- Discontinued intervention **(n=2):**

Sometimes, it’s difficult to catch the foals and take a blood samples

**Analysis**

Analysed (n foals = 42; samples = 674)
Gr**#**1 (**n=114**), Gr**#**2 (**n=113**), Gr**#**3 (**n=117**), Gr**#**4 (n=**110**), Gr**#**5 (n=**107**), Gr**#**6 (**n=113**)
